# Glucagon Shows Higher Sensitivity than Insulin to Grapeseed Proanthocyanidin Extract (GSPE) Treatment in Cafeteria-Fed Rats

**DOI:** 10.3390/nu13041084

**Published:** 2021-03-26

**Authors:** Carme Grau-Bové, Iris Ginés, Raúl Beltrán-Debón, Ximena Terra, MTeresa Blay, Montserrat Pinent, Anna Ardévol

**Affiliations:** MoBioFood Research Group, Department of Biochemistry and Biotechnology, Universitat Rovira i Virgili, 43007 Tarragona, Spain; carme.grau@urv.cat (C.G.-B.); iris.gines@urv.cat (I.G.); raul.beltran@urv.cat (R.B.-D.); ximena.terra@urv.cat (X.T.); mteresa.blay@urv.cat (M.B.); anna.ardevol@urv.cat (A.A.)

**Keywords:** glucagon, GLP1, GLP-1 receptor, procyanidin, insulin/glucagon, cafeteria diet

## Abstract

The endocrine pancreas plays a key role in metabolism. Procyanidins (GSPE) targets β-cells and glucagon-like peptide-1 (GLP-1)-producing cells; however, there is no information on the effects of GSPE on glucagon. We performed GSPE preventive treatments administered to Wistar rats before or at the same time as they were fed a cafeteria diet during 12 or 17 weeks. We then measured the pancreatic function and GLP-1 production. We found that glucagonemia remains modified by GSPE pre-treatment several weeks after the treatment has finished. The animals showed a higher GLP-1 response to glucose stimulation, together with a trend towards a higher GLP-1 receptor expression in the pancreas. When the GSPE treatment was administered every second week, the endocrine pancreas behaved differently. We show here that glucagon is a more sensitive parameter than insulin to GSPE treatments, with a secretion that is highly linked to GLP-1 ileal functionality and dependent on the type of treatment.

## 1. Introduction

The proper functioning of the endocrine pancreas plays a key role in the whole-body energy homeostasis. The concerted actions of insulin and glucagon warrant that plasma glucose levels are kept within a relatively narrow healthy range [1]. An insufficient release of insulin combined with impaired regulation of glucagon secretion is a hallmark of type-2 diabetes [2]. However, there is controversy regarding the relative importance of insulin deficiency and glucagon excess to the hyperglycemia observed in diabetes pathology. Studies with glucagon receptor knockout mice (GlcR−/−) together with streptozotocin-induced destruction of the β-cells indicate that hyperglucagonaemia may be far more important than previously recognized [3]. These results highlight the role played by glucagon in diabetes and indicate that chemical modulation of glucagon release may represent a way of achieving improved glycemic control in diabetes. Indeed, the introduction of treatments based on glucagon-like peptide-1 (GLP-1), which affects both insulin and glucagon secretion [4], illustrates the potential of the α-cells as a pharmacological target.

Glucagon-like peptide-1 (GLP-1) secreted from L-cells of the intestinal tract and from specific cells of the central nervous system exerts pleiotropic biological actions, including the stimulation of glucose-dependent insulin secretion and biosynthesis, inhibition of glucagon secretion, gastric emptying, and inhibition of food intake [5]. GLP-1 shows dysfunction in obesity-related pathologies, such as type 2 diabetes, due to defects in intestinal GLP-1 secretion and β-cell responsiveness [6]. However, α-cells retain near normal responsiveness to GLP-1 infusion, since diabetic and nondiabetic subjects showed similar inhibition of glucagon secretion [7]. The disrupted coordination of glucagon and insulin secretion observed in type 2 diabetes is characterized by impaired and delayed insulin secretion as well as basal hyperglucagonemia and non-suppressed glucagon secretion in response to glucose [8]. It must be highlighted that glucagon receptor knockout mice fed a high-fat diet (HFD) showed better glycemic control and reduced hyperinsulinemia [9]. Furthermore, GLP-1R agonist treatment of HFD mice reversed obesity and insulin resistance [10]. These data highlight that glucagon and GLP-1 have a critical position in the development of hyperglycemia in obese rodents.

It has been shown that an acute dose of grapeseed-derived procyanidin extract (GSPE) can increase plasma GLP-1 [11] and prevent the decrease in GLP-1 associated with a cafeteria diet [12]. Furthermore, this effect is still maintained 17 weeks after GSPE is administered before a cafeteria diet [13]. Some of these effects could be related to the corrective effects of GSPE on β-cells that are disturbed by a cafeteria diet [14]; however, there is no information regarding the effects of GSPE on glucagon production. Here, we show that different GSPE treatments, administered as a preventive treatment against a cafeteria diet according to two different experimental designs, had a stronger effect on glucagon production than on insulin production, altering the insulin/glucagon ratio in different ways depending on the GSPE treatment.

## 2. Materials and Methods

### 2.1. Proanthocyanidin Extract

The grapeseed extracts enriched in proanthocyanidins (GSPE) were kindly provided by Les Dérivés Résiniques et Terpéniques (Dax, France). We used batch numbers 124,029 [13] and 174,860 (containing 21.6% flavan-3-ol monomers, 41.6% dimers+trimers) for the 17-week cafeteria study and the 12-week cafeteria study, respectively.

### 2.2. Animal Experiments

**Figure 1:**
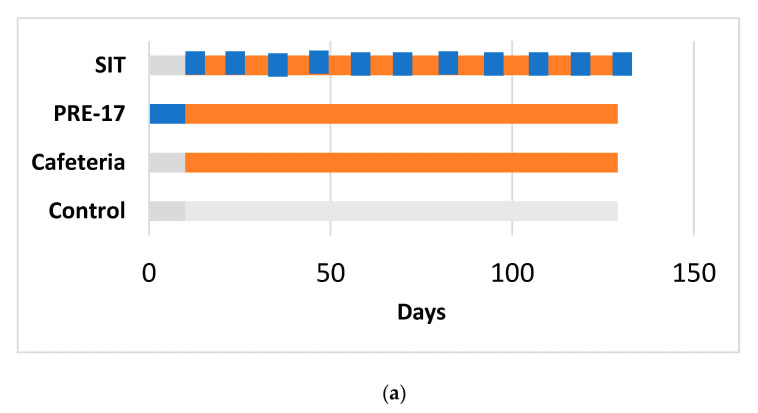

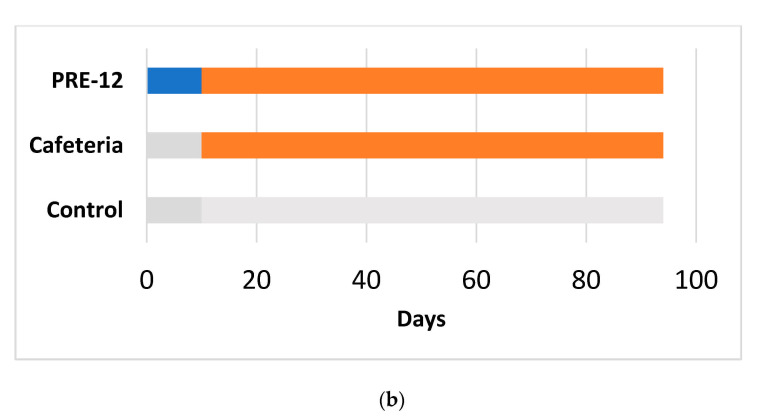
Experimental design for the 17-week (**a**) and 12-week (**b**) cafeteria studies. Control groups received a standard chow diet (grey bar). The other groups received a cafeteria diet (orange bar) for 17 weeks (**a**) or 12 weeks (**b**). The preventive treatment groups (PRE-17) and (PRE-12) received a dose of 500 mg GSPE/Kg (blue bar) for 10 days before starting the cafeteria diet. The simultaneous intermittent treatment-CAF (SIT) group received a five-day dose of 500 mg GSPE/Kg every second week at the same time that they were fed a cafeteria diet.

The animals were kept in animal quarters at 22 °C with a 12-h light/12-h dark cycle and fed ad libitum with a standard chow diet and tap water. We ran two similar experiments (detailed in Figure 1) on female Wistar rats, approved by the Animal Ethics Committee of the Generalitat de Catalunya (respective codes: 0152S/4655/2015 and 10183). 

The 17-week cafeteria study: Rats weighing 240 to 270 g were purchased from Charles River Laboratories (Barcelona, Spain). After one week of adaptation, they were individually caged and randomly distributed into experimental groups (*n* = 7–10/group), as detailed in Figure 1a. The control group received a standard chow diet (Panlab 04, Barcelona, Spain). The other groups received a cafeteria diet for 17 weeks [15] (for detailed composition, see Table S1). At week 14, the food was withdrawn at 10 p.m. The next morning, at 9 a.m., tail blood samples were collected before oral glucose load (2 g of glucose per kg of BW) and 15 min after it. At the end of the study, the animals fasted for 1 to 4 h, were anaesthetized with sodic pentobarbital (70 mg/kg body weight) (Fagron Iberica, Barcelona, Spain), and exsanguinated from the abdominal aorta. Most of the tissues from the animals were immediately frozen in liquid nitrogen and stored at −80 °C for further analysis.

The 12-week cafeteria study: Rats weighing 200 to 225 g were purchased from Envigo (Barcelona, Spain). After one week of acclimation, rats were individually caged and were handled so that they became used to manipulation and oral gavage (twice, with vehicle) during one more week. The animals were separated into three experimental groups (*n* = 10/group), as detailed in Figure 1b. The control group received a standard chow diet (2014-Teklad, Envigo, Barcelona, Spain). The cafeteria group received a cafeteria diet [15] supplemented with 0.5 mL of condensed milk three times a week until the eighth week. After 12 weeks of cafeteria diet, all the animals were overnight fasted and sacrificed by beheading. Most of the animals’ tissues were obtained and immediately frozen in liquid nitrogen and stored at −80 °C until processing. 

### 2.3. Plasma and Tissue Hormone Analysis

The plasma was obtained as previously defined [16]. Pancreatic insulin and glucagon contents were extracted as previously described [17]. Plasma glucose was analyzed using an enzymatic colorimetric kit (GOD-PAP method from QCA, Tarragona, Spain). ELISA kits were used to analyze active GLP-1 and total GLP-1 7–37 amide (EGLP-35K; EZGLP1T-36K, Millipore, Madrid, Spain), insulin and glucagon (10-1251-01; SE-754 50, Mercodia, Uppsala, Sweden), and Amylin (CEA812Ra, Cloud-Clone Corp. Katy, TX, USA). 

The homeostatic model assessment for insulin resistance (HOMA-IR) and the HOMA-β index were calculated using the fasting values of glucose and insulin with the following formulas:

$$\mathrm{HOMA}-\mathrm{IR}=\;\frac{\mathrm{insulin}\;\left(µU/\mathrm{mL}\right)\times \mathrm{glucose}\;\left(\mathrm{mM}\right)}{22.5}$$

$$\mathrm{HOMA}-\beta =\;\frac{20\times \mathrm{insulin}\;\left(µU/\mathrm{mL}\right)}{\mathrm{glucose}\;\left(\mathrm{mM}\right)-3.5}$$

### 2.4. Quantitative Real-Time RT-PCR Analysis 

Total RNA and cDNA were obtained as previously defined [16]. Quantitative PCR amplification was performed using a specific TaqMan probe (Applied Biosystems, Waltham, USA) for the GLP-1 receptor (Rn00562406_m1), proglucagon (Gcg) and the gene encoding for GLP-1 (Rn00562293_m1), and insulin (Rn01774648_g1). The relative expression of each gene was compared with the control group using the 2-∆∆Ct method, with PPIA (Rn00690933_m1) as a reference.

### 2.5. Statistical Analysis

The results are expressed as the mean ± SEM. A Student’s *t*-test was used to compare the treatments with the CAF group. *p*-values < 0.05 were statistically significant. These calculations were performed using the XL-Stat 2017 software (Addinsoft, Paris, France).

## 3. Results and Discussion

### 3.1. Glucagonemia Remains Sensitive to GSPE Treatment Several Weeks after the Treatment Has Finished

Rats that have had a cafeteria diet for 12 weeks have increased glucose and insulin, resulting in increased HOMA-IR and increased HOMA-β compared to the control group (Table 1). These results are in agreement with previous literature that states that the cafeteria diet causes peripheral insulin resistance, and the pancreas compensates for this by producing more insulin [18]. In addition, there is a trend towards higher glucagonemia in a fasting situation (Table 1), as previously described [19]. 

Treatment with 500 mg GSPE /kg BW (PRE-12) for 10 days prior to the 12-week cafeteria diet prevented some of these effects. Table 1 shows that GSPE-treated animals tended to have reduced HOMA-IR, an indicator of peripheral resistance to insulin. The GSPE effects that ameliorate peripheral insulin resistance have been previously described [20]. What is new in this work is the effect of GSPE on glucagon. Under fasting conditions, GSPE-treated animals showed significantly higher plasma glucagon levels than the control group. This trend in glucagon produced a significantly higher glucagon/insulin ratio than in the cafeteria group (Table 1). 

Another group of rats was given the same treatment with GSPE (500 mg/kg BW for 10 days before feeding them a cafeteria diet), but the cafeteria diet was extended to 17 weeks (PRE-17) (Table 2). In this case, these animals were sacrificed under conditions of light fasting, only three hours without food from when the lights were turned on. Under these conditions, neither the cafeteria group nor the GSPE group showed significant differences in plasma glucose or insulin [15]. However, the pre-treatment with GSPE (PRE-17) led to a lower glucagonemia than in the cafeteria group. Furthermore, while the cafeteria group showed a decrease in the insulin/glucagon ratio compared to the standard-fed group, the GSPE pre-treatment normalized this ratio (Table 2). That is, under partially fed conditions at week 17, GSPE pre-treated rats were exposed to an insulin/glucagon signal closer to that of the control group.

### 3.2. Pre-Treatment with GSPE Might Increase GLP-1 Sensitivity in the Pancreas 

We then analyzed the pancreas of the animals. Table 3 shows that 17 weeks of cafeteria diet only produced a trend towards an induced expression of insulin in the pancreas. There were no changes in pancreatic insulin or glucagon. There was a similar situation in the animals treated for 12 weeks with a cafeteria diet. Despite the higher plasma glucagon levels exhibited by the rats that received a pre-treatment with GSPE before the cafeteria diet (PRE-12), these animals did not show statistically significant differences in glucagon content in the pancreas (3846.9 ± 1210, 3433.8 ± 1264, 6239.2 ± 1163 pg glucagon/mg tissue for the control, cafeteria and PRE-12 groups, respectively).

**Figure 2:**
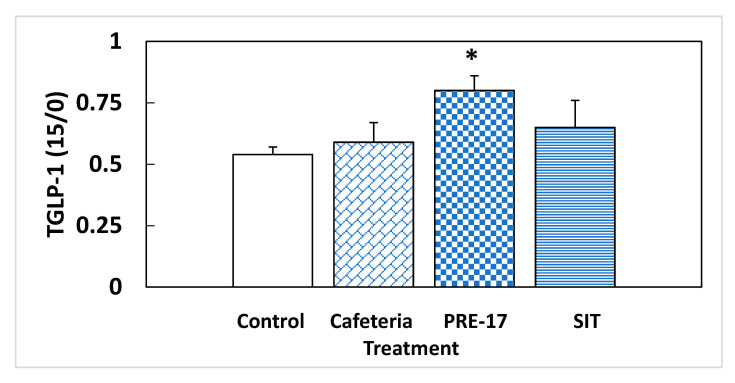
Relative total GLP-1 secretion in a fasting situation. Rats were treated with 0.5 g/Kg BW for the first 10 days, and then they were put on a cafeteria diet for 14 weeks (PRE-17), or a GSPE dose was administered, simultaneously with the cafeteria diet, every second week (SIT). After o/n fasting, a tail blood sample was obtained at time 0 and 15 min after an oral glucose load (2 g/kg BW). At both times, the total GLP-1 was measured, and their ratio was calculated. The data are the mean ± standard error (S.E.M.) (*n* = 7). Statistical differences identified by Student’s *t*-test are defined by * when *p* < 0.05 between treatments.

We analyzed the endocrine pancreas functionality because it is highly influenced by the GLP-1 produced at the intestinal level. At week 17, there were no effects due to the GSPE pre-treatment on plasma-active GLP-1 at sacrifice (5.17 ± 1.98 vs. 3.41 ± 0.46 pM for the PRE-17 and cafeteria groups, respectively). However, we found a different situation in response to an oral glucose load at week 14. The total GLP-1 was measured at time 0 (fasting conditions) and 15 min after oral glucose loading. The change observed between these two points in time was indicative of the new production of GLP-1 due to this glucose load. The cafeteria diet did not show differences in the total GLP-1 ratio (Figure 2). The present results, together with previous data showing no effects of GLP-1 on the ileum or colon of cafeteria-fed rats [13], do not evidence higher intestinal GLP-1 production due to cafeteria treatment. At the pancreatic level, no significant changes were observed in GLP-1R, similar to what was observed in the ileum of the same animals [13] and in the hypothalamus of rats after 12 weeks of cafeteria diet [12]. However, we found a statistically significant increase in total GLP-1 time 15 compared to time zero after a glucose load in the animals that received the GSPE pre-treatment (PRE-17), compared to the cafeteria animals, suggesting a higher ability to secrete GLP-1 after stimulus (Figure 2), although there were no effects on food intake after GSPE treatment at this time point (Table S2 and [21]). This result agrees with the higher GLP-1 expression found in the ileum of these rats [13]. These animals might generate a higher GLP-1 signal after stimulation. This greater GLP-1 signaling was accompanied by a tendency towards an increase in the mRNA expression of GLP-1R in the pancreas, compared to the cafeteria group (Table 3). Therefore, the pancreatic cells of GSPE pre-treated rats are receiving and detecting a higher GLP-1 signal than the cafeteria group when opportunely stimulated. A higher GLP-1 stimulatory signal maintains better pancreatic functioning, together with a higher sensitivity to GLP-1 [22]. The relative presence of GLP-1R in α-cells is very low (70% in β-cells, 60% in δ-cells, and less than 0.5% in α-cells) [22]; however, as shown by Zhang et al. [23], GLP-1R regulation is much more important for glucagon than for insulin. These authors showed that islets of α-cell-specific GLP-1R knockout (αGLP-1R-/-) failed to inhibit glucagon secretion at high glucose levels and failed to stimulate glucagon secretion under very low glucose conditions, with no effects on insulin secretion. Consequently, the trend towards higher GLP-1R maintained 17 weeks after GSPE treatment could be an explanation for the rats’ higher glucagon secretion in fasting situations and their lower secretion in a fed state. 

### 3.3. Administration of GSPE Simultaneously to a Cafeteria Diet Produces Effects on the Endocrine Pancreas That Are Different to the Preventive Approach

Simultaneously to the animals that were fed a cafeteria diet for 17 weeks, we ran an experiment on a group of rats that received treatment with GSPE every second week throughout the cafeteria diet (SIT). 

A different pattern was found in these rats. Under fasting conditions, at week 14 of treatment, there was a trend towards a lower presence of plasma insulin compared to the cafeteria group, with no changes in plasma glucose. This lower insulinemia produced a lower HOMA-IR, although it cannot be read as a lower peripheral resistance due to the high glucose in the fasting situation, which, together with the HOMA-β, evidenced a difficulty to secrete enough insulin to maintain normalized glycaemia (Table 4).

Three weeks later, at the time of death, and in non-fasting conditions, these animals had lower plasma glucagon levels than the cafeteria group. The insulin/glucagon ratio of these animals showed a tendency towards a certain normalization with respect to the cafeteria group (Table 2). They showed more marked effects at the pancreatic level because they showed a tendency to have a lower expression of insulin and glucagon than the cafeteria group, reinforced by a lower amount of glucagon protein contents in this tissue (Table 3). 

We also found that several hours post-stimulation, SIT rats tended to have lower levels of active GLP-1 (2.09 ± 0.46 vs. 3.41 ± 0.46 for the cafeteria group; pM; *p* < 0.1) together with a GLP-1 receptor mRNA expression in the pancreas that was not statistically different from the cafeteria group. The two parameters suggest that the pancreas of the SIT group received lower GLP-1 signaling, which could be an explanation for the limited development found in the endocrine function. This is suggested by the insulin and glucagon mRNA abundance and glucagon contents as well as their limited HOMA-β.

Considering the results at week 14, Figure 2 shows that, at this time, SIT animals had GLP-1 signaling similar to the cafeteria group. We have reported previously that this treatment leads to increased GLP-1 expression in the ileum and colon at week 17. Thus, this lower amount of active GLP-1 in plasma could be a sign of impaired GLP-1 secretion after week 14. Another aspect to be considered is that SIT animals received a similar amount of sucrose as the cafeteria group from the diet, but probably a limited entrance of lipids. GSPE has been shown to be effective for limiting intestinal lipid absorption [24] and these rats showed, at the time of death, lower plasma triglycerides and cholesterol than the cafeteria group [15]. In fact, the food intake of SIT animals was 56% energy derived from sucrose, assuming a total absorption of the of the diet ingested. These animals reproduce a dietary pattern that resembles a high-sucrose diet more than a high-sucrose, high-fat diet, as expected for a cafeteria diet. It has been found that mice receiving a diet with 38.5% sucrose for five weeks showed impairment in GLP-1 secretion [25]. 

## 4. Conclusions

Working with different experimental groups of animals, we show that glucagon is more sensitive than insulin to GSPE. The moment at which the GSPE is administered seems to be a key point in modulating the endocrine pancreas, since preventive and simultaneous treatments induce different endocrine regulations. Without ruling out the possible direct effects of GSPE on the pancreas, the data presented suggest that GSPE has a long-lasting effect on the endocrine pancreas, which is related to the effects of GSPE on GLP-1 ileal production and not related to its effects on food intake. 

## Figures and Tables

**Table 1:** Fasting plasma parameters at the sacrifice of rats treated for 12 weeks with a cafeteria diet with/without a 10-day pre-treatment with grapeseed proanthocyanidin extract (GSPE).

	Control	Cafeteria	PRE-12
Glucose (mM)	6.6 ± 0.2 *	7.56 ± 0.3	7.31 ± 0.4
Insulin (pM)	252.1 ± 27.1 *	589.1 ± 83.0	558.3 ± 111.4
HOMA-IR	9.99 ± 1.2 *	31.89 ± 3.3	22.23 ± 4.2 ^#^
HOMA-β	225.5 ± 27.7 *	387.05 ± 54.88	330.67 ± 48.25
Glucagon (pM)	4.08 ± 1.03 ^#^	9.3 ± 2.44	11.89 ± 2.51 ^§^
Glucagon/insulin	0.024 ± 0.007	0.013 ± 0.0033	0.03 ± 0.007 *

All the data are mean ± SEM of 5–7 animals per group. *t*-tests were applied (* *p* ≤ 0.05 versus cafeteria, ^#^ *p* ≤ 0.1 versus cafeteria; ^§^ *p* ≤ 0.05 versus control). HOMA-IR, homeostatic model assessment for insulin resistance; PRE, preventive treatment groups.

**Table 2:** Plasma and tissue parameters at the sacrifice of rats treated for 17 weeks with cafeteria diet with/without a 10-day pre-treatment with GSPE or a synchronic treatment with GSPE. Animals were sacrificed under a light fast of 3 h.

	Control	Cafeteria	PRE-17	SIT
Glucagon (pM)	7.24 ± 2.28	9.81 ± 1.43	5.83 ± 0.92 *	1.24 ± 1.05 *
Amylin (pg/mL)	8.79 ± 1.10	12.62 ± 2.08	12.91 ± 0.61	9.28 ± 1.86
Insulin/glucagon	0.15 ± 0.02 *	0.07 ± 0.02	0.16 ± 0.03 *	0.10 ± 0.02 ^#^

All the data are mean ± SEM of five to seven animals per group. *t*-tests were applied and are indicated versus the cafeteria group (* *p* ≤ 0.05, ^#^ *p* ≤ 0.1). SIT, simultaneous intermittent treatment.

**Table 3:** Pancreas parameters for rats treated for 17 weeks with a cafeteria diet after a 10-day GSPE pre-treatment (PRE-17) or concomitant to a GSPE treatment every two weeks (SIT).

	Control	Cafeteria	PRE-17	SIT
**Contents in tissue**				
Insulin (ng/g tissue)	59.43 ± 14.88	83.54 ± 15.63	82.48 ± 13.90	51.57 ± 25.44
Glucagon (nmol/g tissue)	787.53 ± 241	917.91 ± 167	958.67 ± 166	304.08 ± 212 *
**mRNA (A.U. vs. Control)**				
Insulin	1.65 ± 0.58 ^#^	6.03 ± 2.35	2.81 ± 0.88	2.17 ± 0.48 ^#^
Glucagon	1.17 ± 0.27	2.88 ± 1.07	0.86 ± 0.48	0.92 ± 0.19 ^#^
GLP-1 Receptor	2.96 ± 1.72	0.86 ± 0.24	2.02 ± 0.47 ^#^	2.71 ± 0.99

Gene expression results are relative to the cafeteria group. All the data are mean ± SEM of five to seven animals per group. *t*-tests were applied and are indicated versus the cafeteria group (* *p* ≤ 0.05, ^#^ *p* ≤ 0.1).

**Table 4:** Fasting plasma samples obtained at week 14 of the rats treated with GSPE simultaneously to a cafeteria diet (SIT).

	Control	Cafeteria	SIT
Glucose (mM)	5.83 ± 0.28 *	7.12 ± 0.12	7.16 ± 0.30
Insulin (μg/L)	0.24 ± 0.03 ^#^	0.39 ± 0.10	0.16 ± 0.002 ^#^
HOMA-IR	1.34 ± 0.12 *	2.01 ± 0.25	1.24 ± 0.05 *
HOMA-β	47.17 ± 1.54	38.99 ± 3.72	21.95 ± 1.86 *

All the data are mean ± SEM of 5-7 animals per group. *t*-tests were applied and are indicated versus the cafeteria group (* *p* ≤ 0.05, ^#^ *p* ≤ 0.1).

## Data Availability

The data presented in this study are available on request from the corresponding author. The data are not publicly available due to lack of platform to publish it.

## Supplementary Materials

The following are available online at https://www.mdpi.com/article/10.3390/nu13041084/s1, Table S1: Cafeteria diet offered to each rat, Table S2: Mean food intake during the five initial days on cafeteria diet.
