# Supplementary material for: Glucagon Shows Higher Sensitivity than Insulin to Grapeseed Proanthocyanidin Extract (GSPE) Treatment in Cafeteria-Fed Rats
Source: Nutrients. 2021 Mar 26;13(4):1084. doi: 10.3390/nu13041084 (PMC8066734; doi:10.3390/nu13041084)
Supplement: Supplementary file 1 [file nutrients-13-01084-s001.pdf]

Table S1. Cafeteria diet offered to each rat:

Food	Amount Offered	Energy Content (kJ)
Bacon	20 g	292.04
Biscuit	6 g	110.46
Paté	4 g	26.28
Muffins	15 g	281.35
Sausages	5 g	52.30
Carrot	10 g	16.48
Milk	125 mL	342.04
Sugar	27.5 g	460.24
Chow	25 g	303.34
Condensed milk	0.5 mL	6.79

Table S2. Mean Food intake during the five initial days on cafeteria diet:

Treatments (Seventeen Weeks Study)	Food Intake (kJ)	Carbohydrate Intake (g)	Lipid Intake (g)	Protein Intake (g)
Control	189.4 ± 8.7 *	7.53 ± 0.34 *	0.50 ± 0.02 *	2.59 ± 0.12 *
Cafeteria	349.8 ± 21.9	14.84 ± 2.06	4.55 ± 0.29	3.87 ± 0.42
PRE-17	333.8 ± 17.0	12.27 ± 0.77	4.30 ± 0.36	2.90 ± 0.24 #
SIT	330.2 ± 12.3	14.96 ± 1.15	4.35 ± 0.17	3.73 ± 0.15
**Treatments (Twelve Weeks Study)**	**Food Intake (kJ)**	**Carbohydrate Intake (g)**	**Lipid Intake (g)**	**Protein Intake (g)**
Control	184.9 ± 10.6*	7.62 ± 0.27 *	0.51 ± 0.02 *	2.63 ± 0.09 #
Cafeteria	438.4 ± 41.6	17.33 ± 3.15	4.52 ± 0.43	3.51 ± 0.39
PRE-12	404.4 ± 38.8	14.57 ± 1.38	4.90 ± 0.49	3.53 ± 0.19

All the data are mean ± SEM of 7–10 animals per group. T-tests were applied (* $p \leq 0.05$ versus Cafeteria; # $p \leq 0.1$ versus Cafeteria)
